# Therapeutic potential of living donor liver transplantation from heterozygous carrier donors in children with propionic acidemia

**DOI:** 10.1186/s13023-022-02233-9

**Published:** 2022-02-21

**Authors:** Zhi-Gui Zeng, Guang-Peng Zhou, Lin Wei, Wei Qu, Ying Liu, Yu-Le Tan, Jun Wang, Li-Ying Sun, Zhi-Jun Zhu

**Affiliations:** 1 Liver Transplantation Center, National Clinical Research Center for Digestive Diseases, Beijing Friendship Hospital, Capital Medical University, No. 101 Lu Yuan Dong Road, Tong-Zhou District, Beijing, 101100 China; 2 Department of Critical Liver Diseases, Liver Research Center, Beijing Friendship Hospital, Capital Medical University, Beijing, 101100 China; 3 Clinical Center for Pediatric Liver Transplantation, Capital Medical University, Beijing, 101100 China

**Keywords:** Propionic acidemia, Living donor liver transplantation, Heterozygous carrier, Propionyl-CoA carboxylase

## Abstract

**Background:**

Current world experience regarding living donor liver transplantation (LDLT) in the treatment of propionic acidemia (PA) is limited, especially in terms of using obligate heterozygous carriers as donors. This study aimed to evaluate the clinical outcomes of LDLT in children with PA.

**Methods:**

From November 2017 to January 2020, 7 of the 192 children who underwent LDLT at our institution had been diagnosed with PA (median age, 2.1 years; range, 1.1–5.8 years). The primary indication for transplantation was frequent metabolic decompensations in 6 patients and preventative treatment in 1 patient. Of the seven parental living donors, six were genetically proven obligate heterozygous carriers.

**Results:**

During a median follow-up of 23.9 months (range, 13.9–40.2 months), all patients were alive with 100% allograft survival, and no severe transplant-related complications occurred. In the case of liberalized protein intake, they did not suffer metabolic decompensation or disease-related complications and made progress in neurodevelopmental delay and body growth, as well as blood and urinary metabolite levels. In one patient with pre-existing mild dilated cardiomyopathy, her echocardiogram results completely normalized 13.8 months post-transplant. All living donors recovered well after surgery, with no metabolic decompensations or procedure-related complications. Western blotting revealed that the hepatic expressions of PCCA and PCCB in one of the heterozygous donors were comparable to those of the normal healthy control at the protein level.

**Conclusions:**

LDLT using partial liver grafts from asymptomatic obligate heterozygous carrier donors is a viable therapeutic option for selected PA patients, with no negative impact on donors’ and recipients' clinical courses.

## Introduction

Propionic acidemia (PA; Online Mendelian Inheritance in Man [OMIM] #606054) is an ultrarare autosomal-recessive disorder of metabolism characterized by biallelic pathogenic variants on chromosome 13q32.3 (*PCCA*) or 3q22.3 (*PCCB*), resulting in the deficiency of the mitochondrial enzyme propionyl-CoA carboxylase (PCC) [1]. Dysfunction of PCC fails to convert propionyl-CoA to methylmalonyl-CoA, thereby leading to the chronic accumulation of propionic acid and propionyl-CoA-related metabolites. These circulating toxic metabolites continuously cause damage to various organs and tissues throughout the body [2]. Since compound heterozygotes in most patients result in undefined genotype–phenotype correlation, affected individuals present with a broad spectrum of clinical manifestations, and age of onset ranges from the neonatal period to adulthood [3]. Characteristic patients usually present in infancy with poor feeding and episodic vomiting within the first few hours to days of life. Without prompt diagnosis and treatment, the illness can progress rapidly to severe ketoacidosis, hyperammonemia, and hyperlactatemia, manifesting as lethargy, seizure, or coma that can result in early death [4, 5]. Despite good compliance with long-term conservative management consisting of individualized nutritional intervention, levocarnitine supplementation, and oral metronidazole, the overall prognosis of PA patients remains poor [6]. Patients surviving their initial metabolic decompensation episode may suffer frequent metabolic decompensations and disease-related long-term multiorgan sequelae such as growth impairment, neurocognitive deficits, cardiomyopathy, pancreatitis, or chronic kidney disease [7, 8]. Furthermore, PA patients' lifelong high-intensity medical and dietary management demands can severely affect family life and cause tremendous financial and psychosocial stress to patients' caregivers [9, 10].

Since replacing the enzyme-deficient liver with a metabolically normal liver could help regain partial PCC activity, after the first attempt in 1991, liver transplantation (LT) has emerged as a novel therapeutic option for selected PA patients who, despite strict dietary and medical intervention, still experience frequent metabolic decompensations or cardiomyopathy [11–15]. In some centers, early LT has been offered as a preventative treatment for metabolically stable pediatric PA patients without metabolic decompensation and severe neurological or cardiac sequelae [13, 14, 16].

Living donors, especially in countries with limited availability of deceased donors, are an essential source of donor organs. And parents of liver transplant candidates are often the predominant and even sole source of living donors. Thus, living donor liver transplantation (LDLT) using an allograft from a heterozygous carrier donor in autosomal-recessive disorders is always inevitable. However, there remains a concern for the potentiality of the insufficient PCC activity in the donor remnant liver and the partial liver graft, and the current world experience of using obligate heterozygous carriers in LDLT for PA patients is limited. Here, we report our single-center experience with LDLT in 7 children with PA, of whom 6 received partial liver grafts from genetically proven heterozygous parental living donors.

## Materials and methods

### Data collection

Patients with PA who underwent LT at our hospital between November 2017 and January 2020 were identified from the liver transplant recipient database. We retrospectively collected demographic data (gender, age, height, weight, age and features of initial clinical manifestations, age at diagnosis, daily protein intake, and medical therapy); transplantation details (indication for LT, age at LT, donor type, graft type, duration of intensive care unit and hospital stay, and postoperative LT-related complications); and laboratory measures (molecular genetic testing, urine organic acid, blood amino acid analysis, blood acylcarnitine profile, renal function, and echocardiography). The neurodevelopmental delay was assessed using the developmental quotient (DQ), which was calculated using the Griffiths Development Scales-Chinese (GDS-C) and the Wechsler Intelligence Scale for Children-IV (WISC-IV) [17]. The hepatic expressions of PCCA and PCCB were assessed at the protein level by western blotting using antibodies directed against PCCA and PCCB proteins (anti-PCCA rabbit polyclonal antibody (ab154254), anti-PCCB rabbit polyclonal antibody (ab96729), Abcam).

### Statistical analysis

Results were expressed as mean ± standard deviation or median (range). Paired sample Student’s *t* test was used for comparative analysis of results before and after transplantation, and *P* < 0.05 was considered a significant difference. Statistical analyses were performed using SPSS software 26.0 (SPSS, Inc., Chicago, IL, USA).

This study was reviewed and approved by the Ethical Committee of Hospital (No. 2020-P2-094-01) and conducted according to the ethical guidelines of the Declaration of Helsinki and the Declaration of Istanbul. We declare that all cases of LDLT were approved by the Ethical Committee of the Hospital, and all living donors were voluntary and altruistic.

## Results

### Patient characteristics

**Fig. 1:**
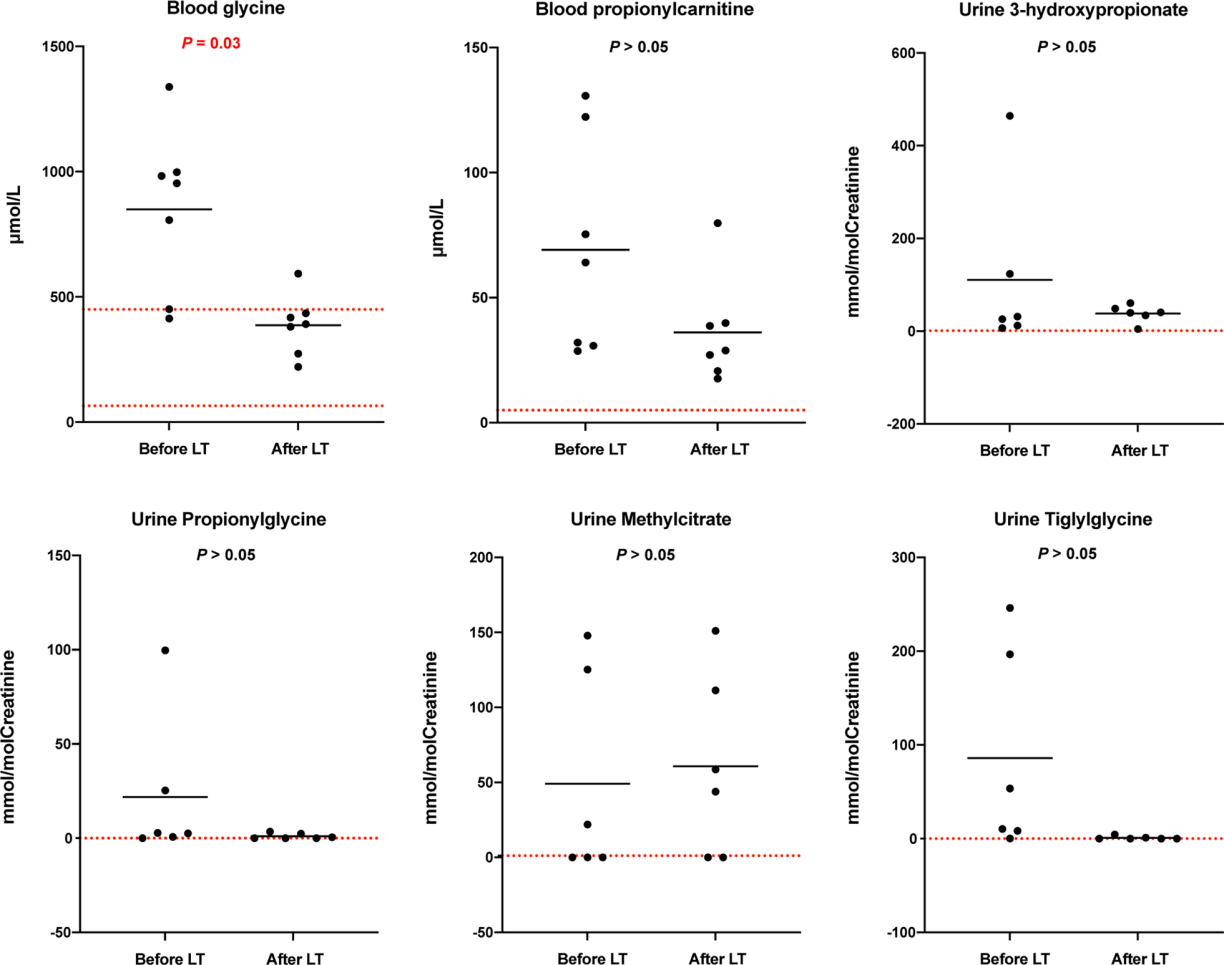
Blood and urinary propionate metabolites levels in PA patients before and after liver transplantation. **A** Blood propionyl carnitine level, **B** blood glycine level; **C** urine 3 OH-propionate; **D** urine propionyl glycine, **E** urine methyl citrate, **F** urine tiglylglycine

Between November 2017 and January 2020, 192 children underwent LDLT at our institution, of whom 7 (3.7%) were diagnosed with PA. According to the age at initial presentation, 3 patients were early-onset (within the first month of life), and the remaining 4 were late-onset (after the first month). The diagnosis of PA was confirmed through a combination of clinical manifestations, typical biochemical findings, and identification of pathogenic variants in *PCCA* or *PCCB* on molecular genetic testing (Table 1; Fig. 1). No patients were from consanguineous parents or had a previously affected relative. After diagnosis, all patients followed a strict protein-restriction diet, a special formula that contains no isoleucine, methionine, threonine, and valine, and were administered levocarnitine supplementation. Before LT, the median protein restriction was 1.75 g/kg/day (range, 0.8–2.7 g/kg/day), and none required supportive feeding (Table 2).

**Table 1:** Demographics, preoperative characteristics, and operative findings of seven recipients with propionic acidemia who underwent LDLT

	Sex	Age at onset/diagnosis	Genetic testing	Initial clinical manifestations	Age at LT, years	Indication for LT	Donor	Heterozygous donor	Graft type	Graft weight, g	GRWR, %	Operation time, minutes	Blood loss, ml/kg	CIT, minutes	ICU Stay, days	Hospital stays, days	Posttransplant complications
1	F	3 days/5 months	c.839dupT, c.996_998del in *PCCA*	Poor feeding, episodic vomiting, lethargy	1.7	Frequent MDs	Mother	Yes (c.996_998del)	Left lateral	210	1.84	500	17.5	145	4	33	CMV Viremia, EBV Viremia
2	F	6 months/7 months	c.30_39del10, c.331C > T in *PCCB*	Poor feeding, lethargy	2.1	Frequent MDs	Father	Yes (c.30_39del10)	Left lateral	299	2.60	390	9.6	100	4	32	CMV Viremia, EBV Viremia, Bile leakage
3	M	6 months/7 months	c.733G > A in PCCB*	Poor feeding, lethargy, muscle hypotonia	5.8	Frequent MDs	Mother	No	Left lateral	252	1.68	525	4.7	85	5	30	EBV Viremia
4	M	3 months/4 months	c.1207C > T, c.319G > A in *PCCB*	Poor feeding, episodic vomiting, lethargy	1.3	Frequent MDs	Father	Yes (c.319G > A)	Reduced-size left lateral	144	1.92	385	40.0	110	6	32	EBV Viremia
5	F	10 days/8 months	c.2002G > A (homozygous) in *PCCA*	Poor feeding, episodic vomiting	2.7	Frequent MDs and dilated cardiomyopathy	Mother	Yes (c.2002G > A)	Left lateral	206	1.87	290	13.6	104	4	12	CMV Viremia
6	M	6 months/8 months	c.331C > T, c.493C > T in *PCCB*	Neurodevelopmental delay	4.5	Preventative treatment	Father	Yes (c.331C > T)	Left lateral	236	1.31	443	5.6	144	2	13	CMV Viremia
7	M	21 days/1.5 months	c.1283C > T, c.839delT in *PCCB*	Poor feeding, episodic vomiting, lethargy, seizure	1.1	Frequent MDs	Father	Yes (c.839delT)	Left lateral	205	1.99	336	8.7	138	13	17	CMV Viremia

*CIT* cold ischemia time, *CMV* cytomegalovirus, *d* day, *EBV* Epstein-Barr virus, *ICU* intensive care unit, *GRWR* graft-to-recipient weight ratio, *LDLT* living donor liver transplantation, *MDs* metabolic decompensations

*There was suspected heterozygous deletion mutation in exon 9 of the *PCCB* gene

**Table 2:** Pretransplant and posttransplant clinical index of patients with propionic acidemia

	Follow-up time, months	Outcome	Current age, years	Metabolism correcting medication		Protein intake, g/kg/day		Support feeding		Metabolic decompensations		Cardiomyopathy		DQ	
				Pre	Post	Pre	Post	Pre	Post	Pre	Post	Pre	Post	Pre	Post
1	40.2	Alive	5.0	Levocarnitine	Levocarnitine	N/A	Normal diet	N	N	Y	N	N	N	77	85
2	39.8	Alive	5.3	Levocarnitine	Levocarnitine	1.25	Normal diet	N	N	Y	N	N	N	69	74
3	37.5	Alive	8.9	Levocarnitine Sodium bicarbonate	Levocarnitine	2.5	Normal diet	N	N	Y	N	N	N	54	57
4	23.9	Alive	3.2	Levocarnitine	Levocarnitine	0.8	Normal diet	N	N	Y	N	N	N	37	45
5	21.6	Alive	4.5	Levocarnitine Sodium benzoate Sodium bicarbonate	Levocarnitine	2.25	Normal diet	N	N	Y	N	Y	N	86	90
6	18.6	Alive	6.1	Levocarnitine	Levocarnitine	2.7	Normal diet	N	N	Y	N	N	N	92	94
7	13.9	Alive	2.3	Levocarnitine	Levocarnitine	1.0	Normal diet	N	N	Y	N	N	N	78	78

*DQ* developmental quotient, *N* no, *Y* yes

### Living donor liver transplantation

Patients underwent LDLT at a median age of 2.1 years (range, 1.1–5.8 years). The indications were frequent metabolic decompensations (6 patients) and preventative treatment (1 patient). All liver grafts were left lateral segments except for one reduced-size left lateral segment, voluntarily donated by their parents. Of the seven parental living donors, six were genetically proven obligate heterozygous carriers (Table 1). All recipients and donors underwent a smooth operation with stable intraoperative metabolic status. Immediate postoperative courses were uneventful, with no metabolic or hepatic decompensations episodes. The median intensive care unit and hospital stays were 4 days (range, 2–6 days) and 30 days (range, 12–33 days), respectively. Immunosuppressive therapy was based on tacrolimus and low-dose corticosteroids. Tacrolimus administration was started the day after LT, and the dose of tacrolimus was dynamically adjusted according to the target whole-blood trough level. The dose of steroids was gradually tapered and withdrawn within the first 6 months after transplantation. With a median follow-up of 23.9 months, all the recipients were alive with 100% allograft survival. Meanwhile, all living donors were discharged from the hospital, recovered well, and reported good working ability after surgery, with no procedure-related complications or metabolic acidosis during the perioperative period and throughout the follow-up period.

**Fig. 2:**
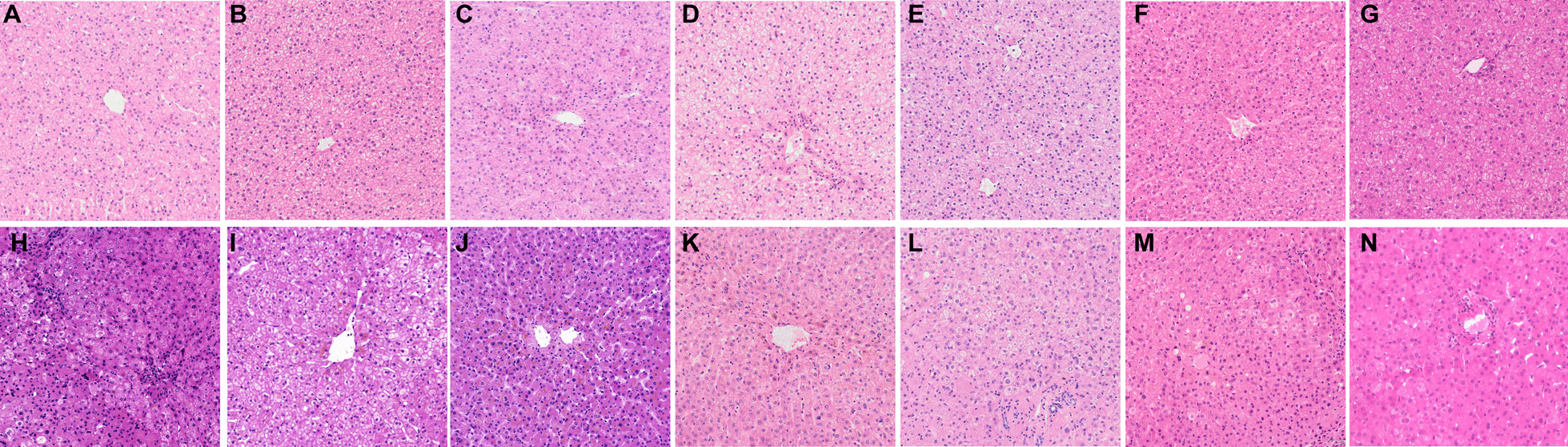
The seven recipients' representative histological finding (H&E staining, 100 ×) of the explanted diseased livers (**A**–**G**) and implanted donor livers (**H**–**N**)

H&E staining of the explanted diseased livers revealed normal liver lobules with the central vein, normal hepatic sinusoid, slightly loose and swollen hepatocytes, no hepatocyte necrosis or vacuolar degeneration, no fibrous tissue proliferation, with a few lymphocytes infiltrated in the partial portal area (Fig. 2A–G). The histopathological examination of the implanted donor livers indicated normal liver lobules with the central vein, normal hepatic sinusoid, and no hepatocyte necrosis or degeneration, with no or mild hepatocyte steatosis (Fig. 2H–N).

### Postoperative transplant-related complications

No recipients developed any vascular complications after LT. A biliary leak was diagnosed in Patient 2, and she was managed conservatively. There was no occurrence of acute or chronic rejection in all 7 recipients during the follow-up period. Cytomegalovirus viremia and Epstein-Barr virus viremia were the most frequent infectious complications, which occurred in 5 and 4 patients, respectively. They all were treated with intravenous or oral ganciclovir. No patient developed suspected or confirmed posttransplant lymphoproliferative disease (PTLD).

### Postoperative metabolic consequences and diet

There was no occurrence of metabolic decompensation in all 7 recipients during the follow-up period, allowing for progressive dietary protein liberalization without the need for any supplements other than levocarnitine. At the last follow-up, all patients had no formal protein restriction.

As part of the routine examination for patients with PA, propionate metabolites in blood [glycine and propionylcarnitine (C3)] and urine (3-hydroxypropionate, propionylglycine, methylcitrate, and tiglylglycine) were measured to evaluate metabolic improvement after LT. All seven patients, at least once, had blood glycine and C3 levels recorded before and after LT. The mean blood glycine level was 849.17 ± 326.59 μmol/L before LT and significantly decreased to 387.36 ± 119.83 μmol/L after LT. This reduction of 461.81 μmol/L reached a statistical significance (*P* = 0.028), and the post-transplant mean glycine levels remained below the normal reference range (65.0–450.0 μmol/L). The mean values of blood C3 reduced from 69.12 ± 43.14 μmol/L before LT to 36.10 ± 21.00 μmol/L after LT. This reduction of 33.02 μmol/L did not reach a statistical significance (*P* = 0.17), and the post-transplant mean C3 levels remained above the normal reference range (1.00–5.00 μmol/L) (Fig. 1).

Six patients had, at least once, urine organic acid assay before and after LT. Compared with pretransplant values, urine 3-hydroxypropionate, propionylglycine, and tiglylglycine mean levels decreased to nearly normal levels after LT, while mean urine methylcitrate increased from 49.17 ± 68.59 mmol/mol Creatinine before LT to 60.81 ± 60.64 mmol/mol Creatinine after LT. However, this increase of 11.64 mmol/mol Creatinine did not reach a statistical significance (*P* = 0.61), and the post-transplant mean methylcitrate levels remained above the normal reference range (0–1.00 mmol/mol Creatinine) (Fig. 1).

### Postoperative renal, cardiac, and neurological consequences

**Fig. 3:**
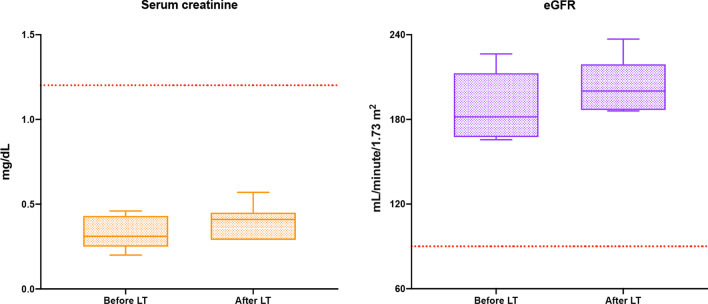
Serum creatinine and estimated glomerular filtration rate (eGFR) before and after liver transplantation

Renal function was evaluated by serum creatinine and estimated glomerular filtration rate (eGFR) calculated according to simplified diet modification in renal disease equation. Values of serum creatinine and eGFR were both within the normal ranges in all patients before and after LT (Fig. 3).

As part of regular assessment, echocardiography was performed for all PA patients during the pretransplant evaluation and every 6 months to 1 year during the post-transplant follow-up. Pre-LT echocardiography was normal in all patients except for Patient 5. She displayed a mild left ventricular dilation and hypokinesia, and the echocardiogram results completely normalized with improved cardiac dilatation and left ventricular function 13.8 months following her transplant. Post-transplant routine echocardiography of other patients was normal.

The neurodevelopmental evaluation using DQ indicated that all patients suffered delayed neurological development prior to LT. Notably, the postoperative DQ levels improved or remained stable in all recipients (Table 2). Despite the improvements in neurodevelopmental delay, all our subjects still did not reach the values appropriate for their age.

### Postoperative body growth

**Fig. 4:**
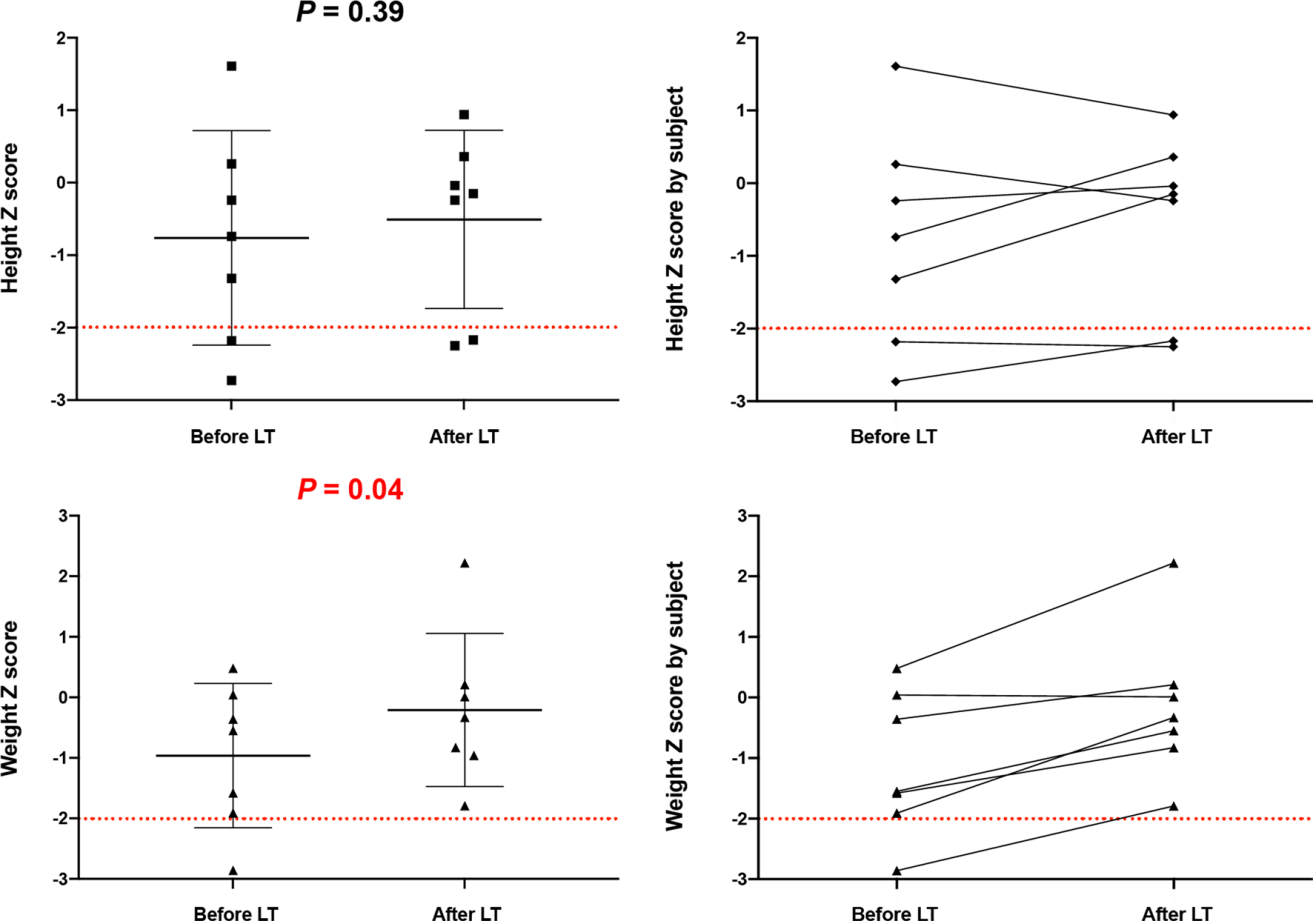
Physical growth parameters (height and weight Z scores) before and after liver transplantation

Body growth parameters, namely height and weight Z scores, were collected before and after LT. There were trends indicating an increase in height Z scores (pre-LT, − 0.76 ± 1.48; post-LT, − 0.51 ± 1.23; *P* = 0.39) and a significant difference between preoperative and postoperative values were observed in weight Z scores (pre-LT, − 0.96 ± 1.19; post-LT, − 0.21 ± 1.26; *P* = 0.04) (Fig. 4).

### The hepatic expressions of PCCA and PCCB in the heterozygous living donor

**Fig. 5:**
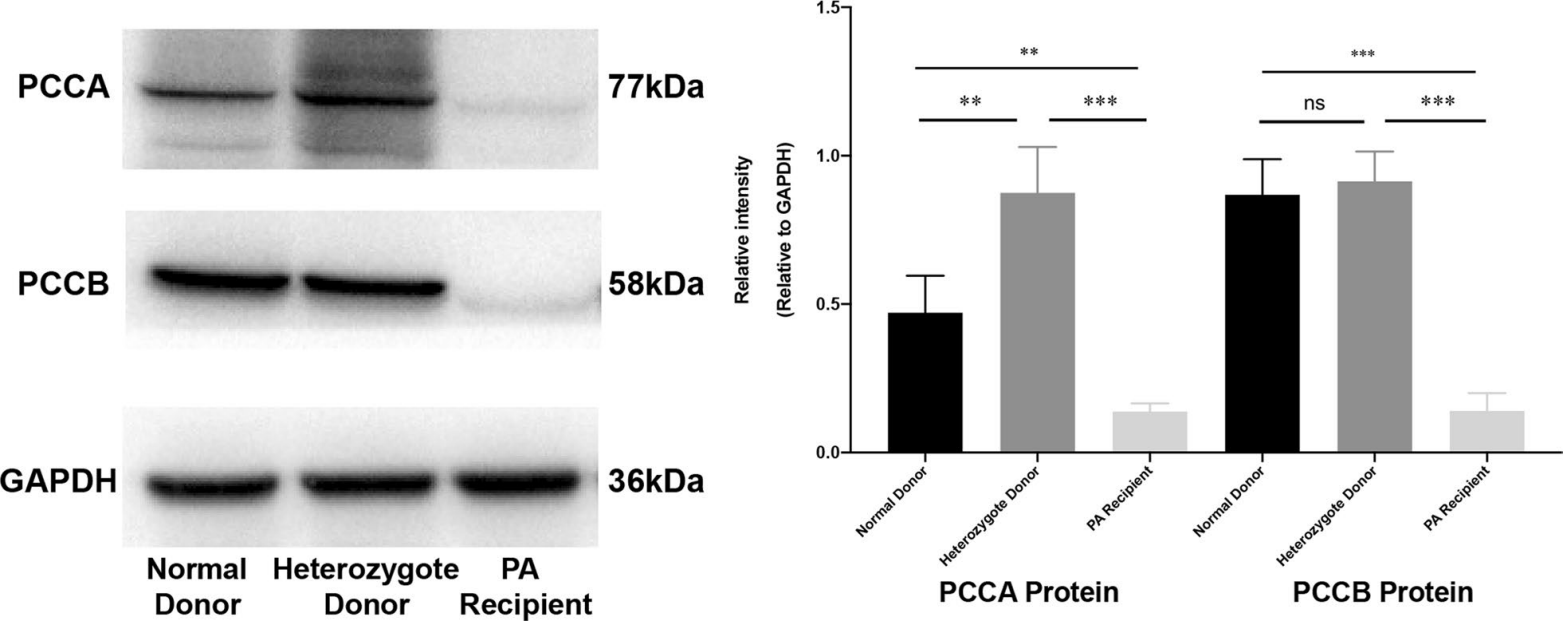
Western blotting using antibodies directed against PCCA and PCCB proteins demonstrate very low hepatic levels of PCCA and PCCB in the PA patient (Patient 7, whose mutation is c.1283C > T, c.839delT in PCCB). The hepatic PCCA expression in the heterozygous donor (Donor 7) was higher than that of the healthy control, while PCCB expression levels were comparable. For all statistical tests, ***p < 0.001 and **p < 0.01 were considered significant. *ns* not significant

The hepatic expressions of PCCA and PCCB in one of the heterozygous parental living donors (Donor 7) were assessed at the protein level and compared to the liver specimens obtained from the PA patient (Patient 7) and a healthy donor. Western blotting using antibodies directed against PCCA and PCCB proteins demonstrated very low levels of PCCA and PCCB in the PA patient’s liver. However, the hepatic PCCA expression in the heterozygous donor was higher than that of the normal control (*P* < 0.01), while the levels of PCCB were comparable (*P* > 0.05) (Fig. 5).

## Discussion

To our knowledge, our series of 7 pediatric PA patients treated with LDLT is the largest to date from a single center. Of the 7 parental living donors, 6 were genetically proven obligate heterozygous carriers. In our report, all 7 recipients achieved satisfactory clinical outcomes with 100% patient and graft survival. There was no occurrence of metabolic decompensation, disease-associated long-term extrahepatic sequelae, or severe LT-related complications during a median follow-up period of 23.9 months. Meanwhile, all living donors recovered well after surgery, without metabolic acidosis or procedure-related complications during the immediate and long-term postoperative periods. More importantly, for the first time, we found that at the protein level, the hepatic expressions of PCCA and PCCB in the heterozygous donor were comparable to the healthy donor.

PA is a rare inborn error of mitochondrial metabolism with life-threatening consequences and multiorgan pathology. Despite following a strict protein restriction and pharmacological intervention, PA patients still suffer frequent metabolic decompensations and subsequent devastating long-term complications [6, 7, 18]. As a kind of enzyme replacement therapy, LT has been performed in 67 PA patients with 75 liver grafts to date [10, 11, 13–15, 19–34]. The overall patient and graft survivals were 80.6% and 72.0%, respectively (Table 3). In the present study, despite a relatively short follow-up of 23.9 months (13.9–40.2 months), our patients' survival outcomes were excellent, with 100% patient and graft survival rates, which were superior to previous data. These improvements may be attributed to the tremendous advances in surgical technology, medical management, and immunosuppressive agents since the 1990s. Hepatic artery thrombosis (HAT), one of the most life-threatening LT-related complications, is more frequent in pediatric LT, with an estimated rate of 4–8% [35]. However, the overall rate of HAT in historical PA cases was significantly high at 17.5% [11]. Therefore, it was once believed that patients with PA are more prone to develop HAT after LT [14, 15]. Contrary to previous results, none of our patients developed HAT post-transplant, and thus we could not confirm a propensity for developing HAT in the liver transplant recipients with PA. Regarding other LT-related complications, neither our study nor previous studies have found that patients with PA were at higher risk. Therefore, concerns about transplant-related complications should not be an obstacle for PA patients to choose LT as a treatment option.

**Table 3:** Literature review of liver transplantation for propionic acidemia

References	n	Onset of PA	Indication for LT	Age at LT (years)	Graft type	Follow-up (months)	Post-LT PA-related complications	Post-LT transplant-related complications	Graft survival	Patient survival
Kayler et al. [34]	1	Early	n/a	3	Deceased	0.3	No	N/A	0/1	0/1
Yorifuji et al. [33]	3	Early (n = 3)	PMC (n = 3)	2.2 (1.2–5.1)	Living (n = 3)	2.5 (1.8–4.9)	MD (n = 1)	N/A	3/3	3/3
Manzoni et al. [32]	1	Early	PMC	0.7	Deceased	0.8	No	N/A	1/1	1/1
Romano et al. [31]	1	Late	CM	6.5	Deceased	0.5	No	N/A	1/1	1/1
Amelook et al. [30]	1	Late	CM	16	Deceased	> 1	No	N/A	1/1	1/1
Kasahara et al. [29]	3	Early (n = 2) Late (n = 1)	PMC (n = 2) CM (n = 1)	2 (0.6–2.2)	Living (n = 3)	1.7 (1.4–3.4)	No	CMV (n = 2)	3/3	3/3
Ryu et al. [28]	1	Early	PMC	1.8	Living	0.01	MD (n = 1)	HVO	0/1	0/1
Arrizza et al. [27]	1	Late	CM	22	Deceased	11	No	N/A	1/1	1/1
Charbit-Henrion et al. [15]	12	Early (n = 12)	PMC (n = 8)^a^ CM (n = 3)^a^ Preventative (n = 2)	3.2 (1.1–9.0)	Deceased (n = 12)	0.39 (0.01–21)	No	BS (n = 1), HAT (n = 4), Primary nonfunction (n = 13), PTLD (n = 2)	5/17	5/12
Honda et al. [26]	1	Early	PMC	4.0	Living	0.13	No	AMR	0/1	0/1
Silva et al. [25]	2	Early (n = 1) Late (n = 1)	CM (n = 2)	12.5 and 5.5	Deceased (n = 2)	4 and 5	No	N/A	2/2	2/2
Critelli et al. [23]	2	Early	CM (n = 1) preventative (n = 1)	8.7 and 1.2	Deceased (n = 2)	2.5 and 1.7	No	ACR (n = 2), CMV (n = 1), HAT (n = 1)	2/2	2/2
Moguilevitch et al. [24]	1	Early	PMC	4.0	Deceased	2	No	N/A	1/1	1/1
Quintero et al. [14]	6	Early (n = 3) Late (n = 3)	PMC (n = 4) Preventative (n = 2)	5.2 (1.3–7.5)	Living (n = 3) Deceased (n = 3)	1.5 (0.5–4.0)	No	HAT (n = 2)	6/6	6/6
Celik et al. [21]	1	Early	PMC	11.8	Living (unrelated)	2.1	No	HAT (n = 1)	1/1	1/1
Chu et al. [10]	2	n/a	n/a	n/a	n/a	n/a	n/a	N/A	2/2	2/2
Pillai et al. [22]	8	Early	PMC (n = 8)	2.0 (0.4–9.4)	Deceased (n = 8)	5.4 (1.3–17.1)	No	ACR (n = 2), Chronic rejection (n = 1), HAT (n = 1), IVC stenosis (n = 1), Portal vein stenosis (n = 1)	8/9	8/8
Shanmugam et al. [20]	5	Early (n = 1) late (n = 1) n/a (n = 3)	PMC (n = 3) CM (n = 1) preventative (n = 1)	2.8 (0.7–4.6)	Living (n = 5)	2.8 (1.6–4.2)	MD (n = 1)	HAT (n = 1)	5/5	5/5
Curnock et al. [13]	14	Early (n = 12) late (n = 1) n/a (n = 1)	PMC (n = 10) preventative (n = 4)	2.4 (0.8–7.1)	Living (n = 1) deceased (n = 13)	4.8 (0.1–22.3)	MD (n = 5) CM (n = 4)	ACR (n = 6), CMV (n = 6), HAT (n = 1), LCR (n = 2), PTLD (n = 1)	11/16	11/14
Tuchmann-Durand et al. [19]	1	Early	PMC and CM	5	Deceased (n = 1)	0.02	n/a	N/A	1/1	1/1

*ACR* acute cellular rejection, *AMR* antibody-mediated rejection, *BS* biliary sepsis, *CM* cardiomyopathy, *HAT* hepatic artery thrombosis, *HVO* hepatic vein obstruction, *LCR* late cellular rejection, *LT* liver transplantation, *MD* metabolic decompensation, *n/a* not available, *PMC* poor metabolic control, *PTLD* posttransplant lymphoproliferative disease

^a^The indication for liver transplantation in one patient was poor metabolic control and cardiomyopathy

Frequent metabolic decompensations are the most common complication of PA patients, leading to frequent hospitalizations and impaired quality of life, and even being life-threatening. Thus, poor metabolic control has become the main indication for LT. Previous studies have demonstrated that LT reduces the risk of metabolic decompensation and improves the quality of life of PA patients [13, 14, 22, 29]. In our study, 6 of the 7 recipients received LT due to frequent metabolic decompensations. Despite not all returning to normal levels, in the case of liberalized protein intake, propionate metabolites in our patients' blood and urine more or less decreased post-transplant. More importantly, no patients suffered further episodes of metabolic decompensation after LT. Therefore, it should be recognized that LT does bring metabolic stability to the already medically fragile PA patients, thereby largely protecting against metabolic decompensation and the need for frequent hospitalizations, which in itself leads to improved quality of life.

Cardiomyopathy, either dilated or hypertrophic, is another common and potentially lethal complication in PA, with an estimated incidence of 9–23% [4, 36]. It also contributes to one of the major causes of mortality in patients with PA [37]. The cardioprotective potential of LT for individuals with PA has been proved that reversal of cardiomyopathy is achieved in all previously reported 11 patients with pre-existing PA-associated cardiomyopathy [15, 20, 23, 25, 27, 29–31]. In line with previous results, one of our patients with pre-existing mild dilated cardiomyopathy displayed a complete recovery of cardiac function after LT. Taken together, LT can be a viable therapeutic option for PA-related cardiomyopathy, and thus severe drug-resistant cardiomyopathy can remain as an indication for LT in patients with PA. Moreover, LT should not be contraindicated in PA patients with severely impaired cardiac function. Devices to stabilize the hemodynamic conditions, such as left ventricular assist device or extracorporeal membrane oxygenation, can be used as a bridge to LT [29, 30].

Neurodevelopmental sequelae are of particular concern in PA patients and the most crucial factor affecting the long-term quality of life. The poor neurocognitive and psychosocial development resulting from metabolic derangement was reported in 43–75% of PA patients [6, 38]. Previous studies suggested that almost all patients presented with neurodevelopmental delay prior to LT made some developmental progress after LT [13, 14, 22]. Our pretransplant neurodevelopmental assessment indicated that all individuals exhibited neurodevelopmental delay. However, LDLT has stabilized each patient's neurological impairment and even improved neurodevelopmental delay to some extent. Nevertheless, neurodevelopmental delay still exists in all surviving patients, and whether sustained neurological improvement could be expected requires more investigations in a longer follow-up.

Sufficient daily intake of essential and functional amino acids is necessary for normal body growth in children [39]. However, PA patients must follow a strict lifelong protein-restricted diet, resulting in severe amino acid deficiencies, so they are prone to develop body growth retardation [40]. A post-transplant liberalized protein diet means sufficient intake of essential and functional amino acids, which is crucial to correct chronic malnutrition and stimulate body growth in liver transplant recipients with PA. In turn, optimal growth leads to higher protein tolerance, which possibly helps further to reduce the risk of metabolic decompensation after transplantation [40]. In our series, in the case of no formal protein restriction, post-transplant mean height and weight Z scores were both improved compared with the pre-transplant levels. However, whether the present patients' physical growth would catch up to the standard growth curve warrants further long-term follow-up.

Given the shortage of available donor organs and low priority in the waiting list, LDLT has been a feasible choice for inborn errors of metabolism, in which the donor is almost always a blood relative of the patient, and parental donors are preferable [41, 42]. Since most monogenetic diseases are inherited in an autosomal recessive fashion, the parent who serves as a living donor is almost always an obligate heterozygous carrier. However, whether using a partial allograft from a heterozygous living donor contributes to sufficient metabolic correction in a homozygous recipient remains a concern. There is a possibility that the implanted partial liver graft may have low PCC activity, leading to persistent insufficient enzyme activity in the recipient, which will compromise the therapeutic value of LDLT in PA. There is also the possibility that the residual liver's PCC activity in the heterozygous donor is insufficient, which may put the donor in danger of disease-related symptoms and complications. Curnock et al. [13] hold the view that it should be preferential to use an unrelated LT donor in patients with PA. In contrast, previous studies suggested that no mortality or morbidity associated with the use of heterozygous carrier donors was observed in donors or recipients [16, 29, 33, 41]. Our study is believed to be the largest reported series of PA patients receiving partial liver grafts from genetically proven obligate heterozygous carriers. We demonstrated satisfactory clinical outcomes in all 6 recipients, with no negative impact on both donors' and recipients' clinical courses. More importantly, for the first time, our study demonstrated that the hepatic expressions of PCCA and PCCB at the protein level in one of the heterozygous parental living donors were equal to those of the healthy donor. These results clearly indicate that LDLT using obligate heterozygous carriers as donors is a viable therapeutic option for PA. An early LT (ideally within the first year of life) has been considered for younger children with non-severe PA [13–15], and scheduled LT, according to the state of the disease, can almost only be performed in the setting of LDLT. Considering the promising therapeutic value of LDLT in the treatment of PA, LDLT using obligate heterozygous carriers as donors could be considered for selected PA patients, especially in countries with a limited deceased donor pool.

Although LT can theoretically provide a lifelong supply of PCC activity within the allograft, successful transplantation does not result in a cure in individuals with PA due to the ubiquitous enzyme deficiency throughout the body. Other extrahepatic tissues with remaining PCC deficiency, such as skeletal muscle, brain, heart, and kidneys, persistently produce pathognomonic propionate metabolites after LT [43]. These circulating toxic metabolites in turn cause damage to the central nervous system, heart, kidneys, and other organs throughout the body. Therefore, continued levocarnitine supplementation in the posttransplant period is advocated for all patients to enhance the excretion of propionate metabolites. Nevertheless, some liver transplant recipients with PA have been reported to develop metabolic decompensation, cardiomyopathy, and kidney dysfunction during long-term follow-up [13–15, 20, 28, 33, 44]. Although our patients did not experience the above-mentioned PA-related complications, it is recognized that LT can only achieve partial clinical improvements of this devastating disease and delay the progression of the disease or complications but cannot completely change the natural history of PA. The possibility of post-transplant non-remission or even chronic progression of the disease, especially the occurrence of long-term disease-related complications, should always be kept in mind by transplant surgeons. And patients' parents or guardians should be fully informed of these potential risks before LT. Lifelong regular follow-up, including metabolic, cardiac, renal, and neurological monitoring and evaluation, is necessary for all individuals with PA post-transplant. An experienced interdisciplinary team consisting of metabolism physicians, pediatric hepatologists, pediatric neurologists, pediatric cardiologists, pediatric nephrologists, pediatric transplantation team, metabolic dieticians, and neurorehabilitation physicians is also essential for the long-term management of patients with PA.

This study's limitations included its single-center retrospective nature, small sample size, and relatively short-term follow-up. Nevertheless, we found for the first time that the hepatic expressions of PCCA and PCCB in the heterozygous parental living donor were equal to those of the healthy donor, which provides a solid foundation for the clinical use of LDLT from obligate heterozygous donors in patients with PA.

## Conclusion

In summary, our study provides evidence that the hepatic expressions of PCCA and PCCB from the heterozygous donor are comparable to those of the healthy control at the protein level. LDLT using partial liver grafts from asymptomatic obligate heterozygous carrier donors is a viable therapeutic option for selected patients with PA, with no negative impact on both donors' and recipients' postoperative courses.

## Data Availability

All data generated or analyzed during this study are included in this published article.

## Abbreviations

ACR: Acute cellular rejection
AMR: Antibody-mediated rejection
BS: Biliary sepsis
CM: Cardiomyopathy
CMV: Cytomegalovirus
DQ: Developmental quotient
EBV: Epstein-Barr virus
eGFR: Estimated glomerular filtration rate
HAT: Hepatic artery thrombosis
HVO: Hepatic vein obstruction
ICU: Intensive care unit
GDS-C: Griffiths Development Scales-Chinese
LT: Liver transplantation
LDLT: Living donor liver transplantation
MDs: Metabolic decompensations
n/a: Not available
PA: Propionic academia
PCC: Propionyl-CoA carboxylase
PMC: Poor metabolic control
PTLD: Posttransplant lymphoproliferative disease
WISC-IV: Wechsler Intelligence Scale for Children-IV
